# Cuscuta chinensis Lam. Protects Against Light-Induced Retinal Degeneration: Therapeutic Implications for Photoreceptor Degenerative Disorders

**DOI:** 10.3389/fphar.2022.904849

**Published:** 2022-06-08

**Authors:** Hanhan Wu, Beijing Zhu, Daijin Li, Jing Xu, Jie Chang, Xiaoye Du, Jingang Cui, Ning Zhang, Teng Zhang, Yu Chen

**Affiliations:** ^1^ Yueyang Hospital of Integrated Traditional Chinese and Western Medicine, Shanghai University of Traditional Chinese Medicine, Shanghai, China; ^2^ Baoshan Hospital of Integrated Traditional Chinese and Western Medicine, Shanghai University of Traditional Chinese Medicine, Shanghai, China; ^3^ Clinical Research Institute of Integrative Medicine, Shanghai Academy of Traditional Chinese Medicine, Shanghai, China; ^4^ Science and Technology Laboratory Center, Shanghai University of Traditional Chinese Medicine, Shanghai, China

**Keywords:** Cuscuta chinensis lam., retina, photoreceptor degeneration, cell death, müller gliosis, microglial activation, retinal inflammation

## Abstract

*Cuscuta chinensis* Lam. (CCL) is a medicinal herb widely used in traditional Chinese medicine for the treatment of ophthalmic diseases, including age-dependent vision-threatening retinal degenerative disorders that involve irreversible loss of the first-order retinal neurons, photoreceptors. However, evidence is lacking if CCL is pharmacologically active at protecting against loss of photoreceptors and photoreceptor degeneration-associated retinal structural and functional impairment. The current study thus evaluates the potential photoreceptor protective effects of CCL to better support its clinical applications in the prevention and treatment of photoreceptor degenerative diseases. Non-invasive full-retinal optical coherence tomography, electroretinography, histological examination, immunohistochemistry and real-time qPCR analysis were performed to assess the retinal protective effects of CCL in light-exposed BALB/c mice characterized by photooxidative stress-mediated photoreceptor loss and associated retinal morphological and functional impairment. The results showed that CCL treatment protected against light-induced degeneration of the photoreceptor structure and deterioration of the retinal function. Furthermore, CCL treatment increased the retinal expression of rhodopsin, S-opsin and M-opsin, supporting the protective effects of CCL in both rod and cone photoreceptors. CCL treatment suppressed photoreceptor cell death in the light-exposed retinas. The morphological integrity of the second-order retinal neurons was also preserved as a result of CCL treatment. In addition, CCL treatment attenuated light-induced reactive müller gliosis, microglial activation and inflammation in the retina. In conclusion, the current work demonstrates for the first time that CCL protects against photooxidative stress-mediated degeneration of photoreceptors and associated disturbance of structural, functional and immune homeostasis of the retina. The findings here thus provide novel experimental evidence supporting the clinical application of CCL in the prevention and treatment photoreceptor degenerative diseases.

## Introduction

Photoreceptors are the first-order retinal neurons that execute the function of light absorption and phototransduction, playing an essential role in the formation of vision. Photoreceptor degeneration constitutes the central pathology that accounts for vision impairment or in the worst case scenario, blindness in patients with retinal degenerative diseases, for instance, age-related macular degeneration (AMD), Stargardt disease, and retinitis pigmentosa (RP) (Curcio et al., 1996; Wenzel et al., 2005). Currently, effective photoreceptor protective therapies are still lacking in Western medicine.

Oxidative stress directly leads to photoreceptor cell death, causing degeneration of the photoreceptor structure and impairment of the retinal function (Carmody et al., 1999; Krishnamoorthy et al., 1999; Carmody and Cotter, 2000; Ryter et al., 2007). Exaggerated inflammatory responses resulting from the initial degenerative changes in the retina further exacerbates photoreceptor death, playing an important role in the progression and deterioration of photoreceptor degenerative disorders (Telander, 2011; Kohno et al., 2013; Chen and Xu, 2015). *Cuscuta chinensis* Lam (CCL) has a long history of the clinical usage as a component of traditional Chinese medicine (TCM) formulas for the treatment of ophthalmic disorders manifesting impaired vision encountered in patients with various ophthalmic diseases including photoreceptor degenerative diseases (Zheng HZD and She, 1998; Zhu et al., 2020). However, TCM-guided clinical application of CCL in the treatment of photoreceptor degenerative disorders is to a large extent empirical, which is primarily based on the symptoms (e.g. dim or blurred vision) instead of pathological mechanisms or disease entities. Meanwhile, pharmacological studies have demonstrated the anti-apoptotic, anti-oxidant and anti-inflammatory activities of CCL in various pathophysiological processes (Donnapee et al., 2014), while the pharmacological implications of CCL in photoreceptor degenerative diseases remain unexplored. It is unknown whether CCL is pharmacologically effective at protecting against the retinal structural and functional impairment caused by photoreceptor degeneration. Clarifying the photoreceptor protective properties of CCL helps to better orient the clinical application of CCL in the prevention and treatment of photoreceptor degenerative diseases.

Bright light-induced retinal degeneration in BALB/c mice is characterized by photooxidative stress-mediated photoreceptor degeneration and ensuing second-order neuronal impairment, reactive müller gliosis, microglial activation and retinal inflammation, recapitulating the hallmark pathologies in patients with photoreceptor degenerative disorders such as AMD, Stargardt disease and RP (Young, 1988; Organisciak et al., 1998). Thus, the protective effects of CCL against photoreceptor degeneration were evaluated in experimental light-exposed BALB/c mice in the current study.

## Materials and Methods

### HPLC Analysis

CCL seeds (Lot No. 190208) were purchased from Shanghai Kangqiao Chinese Medicine Tablet Co., Ltd. (Shanghai, China). For the quality control analysis, ground CCL powder was accurately weighed, dissolved in 80% methanol and extracted by sonication (Power 300 W, Frequency 40 kHz) for 60 min. CCL decoction (2.4 g/ml) was diluted with 80% methanol to a final concentration of 0.48 g/ml. The solution was filtrated through a 0.22-µm filter. 2 μl of solution was injected for HPLC analysis. The stock solutions of the standard compounds neochlorogenic acid (Lot No. 19081403, Chengdu Pufei De Biotech Co., Ltd., China), chlorogenic acid (Lot No. Y20A11K111541, Shanghai Yuanye Bio-Technology Co., Ltd., China), cryptochlorogenic acid (Lot No. 20111206, Chengdu Pufei De Biotech Co., Ltd., China), caffeic acid (Lot No. 20110501, Shanghai Yuanye Bio-Technology Co., Ltd., China), p-coumaric acid (Lot No. 18011605, Chengdu Pufei De Biotech Co., Ltd., China), hyperoside (Lot No. P14A11F121347, Shanghai Yuanye Bio-Technology Co., Ltd., China), Astragaline (Lot No. 20062173, Shanghai Shifeng Biological Technology Co., Ltd., China), quercetin (Lot No. 20071651, Shanghai Shifeng Biological Technology Co., Ltd., China) and kaempferol (Lot No. 20061127, Shanghai Shifeng Biological Technology Co., Ltd., China) were prepared in 80% methanol and further diluted using methanol to a final concentration of 14.5 μg/ml, 55 μg/ml, 18.5 μg/ml, 13 μg/ml, 18.5 μg/ml, 83.4 μg/ml, 28.4 μg/ml, 9.04 μg/ml, and 9.9 μg/ml, respectively. All of the solutions were stored at 4°C until analysis. The content of the major phenolic acids including neochlorogenic acid, chlorogenic acid, cryptochlorogenic acid, caffeic acid, p-coumaric acid and major flavonoids such as hyperoside, astragaline, quercetin and kaempferol in the ground CCL powder and CCL decoction was analyzed using an Agilent HPLC 1200 system (Agilent Technologies, United States). Chromatographic separation was conducted on Symmetry Shield RP18 column (4.6 × 250 mm, 5 μm) at the column temperature of 30°C. The mobile phase for the developed method consisted of 0.1% phosphoric acid in water (solvent A) and acetonitrile (solvent B). The method involved a stepwise linear gradient as follows: 8% solvent B at 0–8 min, 8–14% solvent B at 8–50 min, 14–20% solvent B at 50–65 min, 20–30% solvent B at 65–80 min, 30–50% solvent B at 80–90 min, 50–95% solvent B at 90–95 min, and then 95% solvent B at 95–105 min. In addition, the velocity of flow was 1 ml/min. The detection wavelength was set at 328 nm. Chemstation software (Agilent Technology) was used for peak detection and peak area calculation.

### Animals and Treatments

Four to five-week-old BALB/c mice (20 ± 1.1 g body weight, bw) (Shanghai Laboratory Animal Research Center, China) were maintained under a 12/12 h light/dark cycle with temperature set at 24 ± 2°C. The mean luminance of the animal research core facility is kept at 15–20 lux. CCL decoction at the concentration of 2.4 g/ml and 0.6 g/ml was prepared by boiling and concentrating CCL seeds in water. The mice were dark-adapted for 24 h prior to white light exposure (Compact Fluorescence Lamp, 45 W, Chaoya Lighting, Shanghai, China) delivered at 15,000 lux for 30 min. The mice unexposed to the experimental light were treated with sterile water to serve as the normal controls. Light-exposed mice were intraperitoneally injected with sterile water or a single dose of CCL at 3 g/kg bw (designated as low-dose CCL, CCL-L) and 12 g/kg bw (designated as high-dose CCL, CCL-H or CCL for the indicated analyses) in a controlled volume of 100 μL per mouse 30 min prior to light exposure following the treatment protocols as described in our previous studies (Bian et al., 2016; Chen et al., 2016). All protocols were reviewed and approved by the Institutional Animal Care and Use Committee of Yueyang Hospital of Integrated Traditional Chinese Medicine, Shanghai University of TCM (YYLAC-2019-067) and carried out in adherence to the Association for Research in Vision and Ophthalmology Statement for the Use of Animals in Ophthalmic and Vision Research.

### Optical Coherence Tomography

Image-guided OCT (OCT 2 with Micron IV, Phoenix Research Labs, United States) was performed to obtain full-retinal scans in the live animals as previously described (Wu et al., 2020). Briefly, in preparation for the OCT imaging, anesthesia was induced by intraperitoneal injection of ketamine hydrochloride (82.5 mg/kg bw) and xylazine (8.25 mg/kg bw), which was followed by pupil dilation using 1% tropicamide (Santen Pharmaceutical, Japan). OCT scans acquired and averaged using Phoenix Reveal OCT Software (Phoenix Research Labs, United States) were then subjected to thickness measurement of the outer nuclear layer (ONL) using Insight Image Segmentation Software for the Phoenix OCT and Retinal Imaging System (Version 2.0.5490, Voxeleron LLC, United States).

### Electroretinography

The scotopic ERG responses were measured using Ganzfeld (ERG 2, Phoenix Research Labs, United States) and analyzed by LabScribe software (Phoenix Research Labs, United States) as previously described (Wu et al., 2020). In brief, dark-adapted mice were anesthetized by ketamine hydrochloride and xylazine cocktail as indicated above, followed by pupil dilation and ERG recording under safe-light conditions (5 lux). For ERG recording, flashes of green light (504 nm) were delivered at the intensity of −2 (0.5 msec duration and 5 s inter-stimulus-interval), −0.8 (1 msec duration and 5 s inter-stimulus-interval), 0.4 (1 msec duration and 10 s inter-stimulus-interval), 1.6 (1 msec duration and 20 s inter-stimulus-interval) and 3.1 (1 msec duration and 60 s inter-stimulus-interval) log cd·s·m^−2^.

### Histological and Immunohistochemical Examination

For the making of paraffin sections, eyes were enucleated and fixed in 4% paraformaldehyde and subject to further processing using a Benchtop Tissue Processor (Leica TP1020, Germany). Tissue blocks were then embedded using a paraffin dispenser in conjunction with a cold plate (Leica EG1150H&C Embedding Center, Germany). Paraffin sections in the thickness of 4 μm were cut using a rotary microtome (Leica RM2235, Germany). For the making of cryosections, the cornea and lens were removed from the enucleated eyes and the remaining eye cups were fixed in 4% paraformaldehyde. After washing the fixed eye cups in PBS, manual dehydration was performed using 5, 10, 15 and 30% sucrose solutions, followed by embedding in optimal cutting temperature compound (Tissue-Tek, Sakura Finetek, United States). Cryosections in the thickness of 12 μm were cut using a cryostat (Leica CM3050S, Germany). Gross retinal histology was examined by hematoxylin and eosin (H&E) staining of the paraffin sections and observed using a light microscope (DM 2000, Leica, Germany). The number of the photoreceptor cell nuclei was counted using ImageJ/FIJI (https://fiji.sc/, NIH, United States) as previously described (Byun et al., 2006). Paraffin sections were also examined for the expression of rhodopsin and short wavelength-sensitive cone opsin (S-opsin) and middle wavelength-sensitive opsin (M-opsin) in the retina. Cryosections were examined for the retinal expression of glial fibrillary acid protein (GFAP), PKCα, Calbindin D and Iba-1. Briefly, deparaffinized sections or cryosections were incubated with primary antibodies including mouse anti-rhodopsin antibody (1:1,000, Novus Bio, United States), rabbit anti-opsin S antibody (1:100, Millipore, United States), rabbit anti-opsin M antibody (1:100, Millipore, United States), rabbit anti-protein kinase C α (PKCα) antibody (1:5,000, Sigma, United States), rabbit anti-calbindin D antibody (1:1,000, Abcam, United States), goat anti-GFAP antibody (1:500, Dako, United States), or rabbit anti-Iba1 antibody (1:500, Wako, Japan), which was followed by incubation of secondary antibodies including Cy3-conjugated sheep anti-mouse or sheep anti-rabbit secondary antibodies (1:1,000, Sigma, United States). Counterstaining of 4-6-diamidino-2-phenylindole (DAPI) was performed to visualize the nuclei. The immunoreactivity was observed by a fluorescent microscope (DM6000B, Leica, Germany). Image processing was performed using ImageJ/FIJI in reference to the previously published methods (Carvalho et al., 2018; Akiba et al., 2019).

### TdT-Mediated dUTP Nick-End Labeling (TUNEL) Assay

Cryosections were used for the detection of cell death by a TUNEL assay (DeadEnd™ Fluorometric TUNEL system, Promega, United States) following the manufacturer’s instructions. Briefly, sections were fixed in 4% paraformaldehyde and permeabilized with 20 μg/ml proteinase K solution. Sections were then incubated with equilibration buffer at room temperature for 10 min, which was followed by incubation with TdT reaction buffer at 37°C for 45 min in a humidified chamber. Images were observed by a fluorescent microscope (DM6000B, Leica, Germany).

### Real-Time Quantitative Polymerase Chain Reaction (Real-Time qPCR)

The retinas were collected after the indicated treatment. Total RNA was then isolated usingTRIzol (TaKaRa, Japan) reagent, followed by reverse transcription using the PrimeScriptTM RT reagent Kit (TaKaRa, Japan). Real-time qPCR was subsequently performed with the SYBR Green I Master (Roche, United States) on a Roche Light Cycler 480 II (Roche, United States). The sequences of primers used for real-time qPCR analysis were listed in Table 1. 18S ribosomal RNA (rRNA) was amplified as an internal control. The fold change in the gene expression was calculated based on 2^−[Ct (specific gene)−Ct (18s rRNA)].^

**TABLE 1:** The primer sequences.

Gene name		Primer sequences (5′3′)
Ccl2	Forward primer	AGC​TGT​AGT​TTT​TGT​CAC​CAA​GC
	Reverse primer	GTG​CTG​AAG​ACC​TTA​GGG​CA
Gfap	Forward primer	CCG​AGT​ACT​GAA​GCC​AAG​GG
	Reverse primer	GCA​GTT​TGT​AAC​CCC​TCC​CA
Il1b	Forward primer	TGC​CAC​CTT​TTG​ACA​GTG​ATG
	Reverse primer	AAG​GTC​CAC​GGG​AAA​GAC​AC
Il6	Forward primer	CCA​AGA​ACG​ATA​GTC​AAT​TCC​AGA​A
	Reverse primer	AAG​AAG​GCA​ACT​GGA​TGG​AAG​T
Tnf	Forward primer	ACG​TCG​TAG​CAA​ACC​ACC​AA
	Reverse primer	GCA​GCC​TTG​TCC​CTT​GAA​GA
18S rRNA	Forward primer	GAG​GTT​CGA​AGA​CGA​TCA​GA
	Reverse primer	TCG​CTC​CAC​CAA​CTA​AGA​AC

## Statistical Analysis

All results were expressed as mean ± standard deviation (SD). Statistical analyses were performed using independent-samples *t*-test (GraphPad Prism 8, United States). Differences were considered statistically significant if *p* < 0.05.

## Results

### CCL Treatment Protects Photoreceptors From Developing Light-Induced Structural Impairment

**FIGURE 1:**
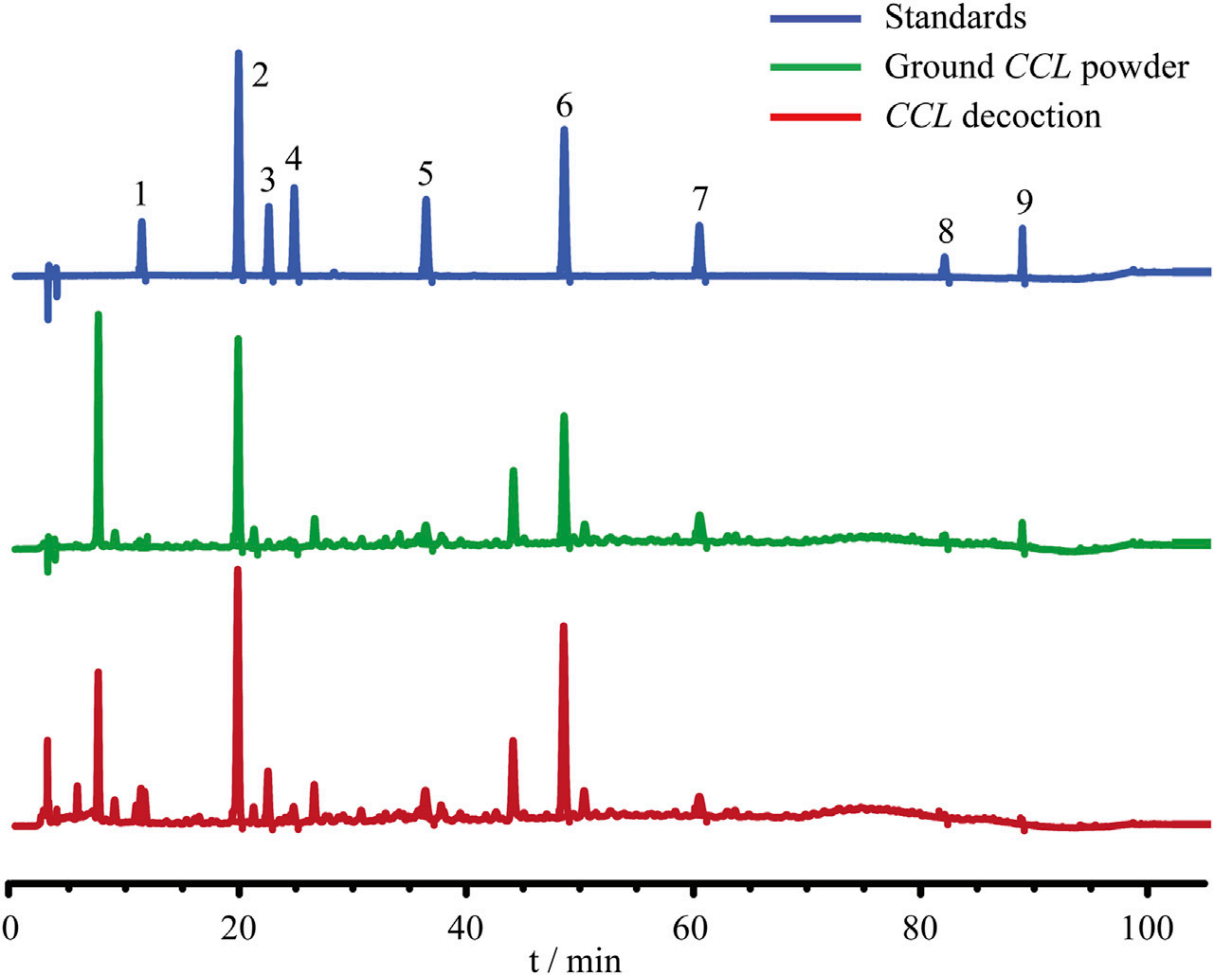
Quality control analysis of CCL. For the quality control purpose, HPLC analysis was performed to estimate the content of neochlorogenic acid, chlorogenic acid, cryptochlorogenic acid, caffeic acid, p-coumaric acid, hyperoside, astragaline, quercetin and kaempferol in ground CCL powder and CCL decoction. The detection wavelength was set at 328 nm. Peak detection and peak area calculation was performed using Chemstation software.

Prior to the indicated analysis, HPLC was performed to obtain the chemical profile of the major components in CCL for the quality control purpose. The results showed that phenolic acids including neochlorogenic acid, chlorogenic acid, cryptochlorogenic acid, caffeic acid, p-coumaric acid and flavonoids including hyperoside, astragaloside, quercetin and kaempferol could be detected in the ground CCL powder and CCL decoction (Figure 1). According to Pharmacopoeia of the People’s Republic of China (2020 Edition), the content of hyperoside, the quality control compound for CCL, should be no lower than 0.01% in the dried CCL. The results from the HPLC analysis showed that the amount of hyperoside in the ground CCL powder was estimated to be approximately 0.1296%, validating the quality of CCL used in the current study. Meanwhile, HPLC analysis also revealed that the major phenolic acids and flavonoids were present in the CCL decoction used for the following assessment (Table 2).

**TABLE 2:** Estimated content of the major chemical components in CCL.

Compound	Estimated content (%)^a^	Estimated content (%)^b^
Neochlorogenic acid	0.0069	0.0029
Chlorogenic acid	0.1653	0.0149
Cryptochlorogenic acid	0.0123	0.0031
Caffeic acid	0.0047	0.0009
p-Coumaric acid	0.0221	0.0023
Hyperoside	0.1296	0.0145
Astragaline	0.0350	0.0022
Quercetin	0.0053	0.0002
Kaempferol	0.0104	0.0001

a Ground CCL powder was analyzed.

b CCL decoction was analyzed.

**FIGURE 2:**
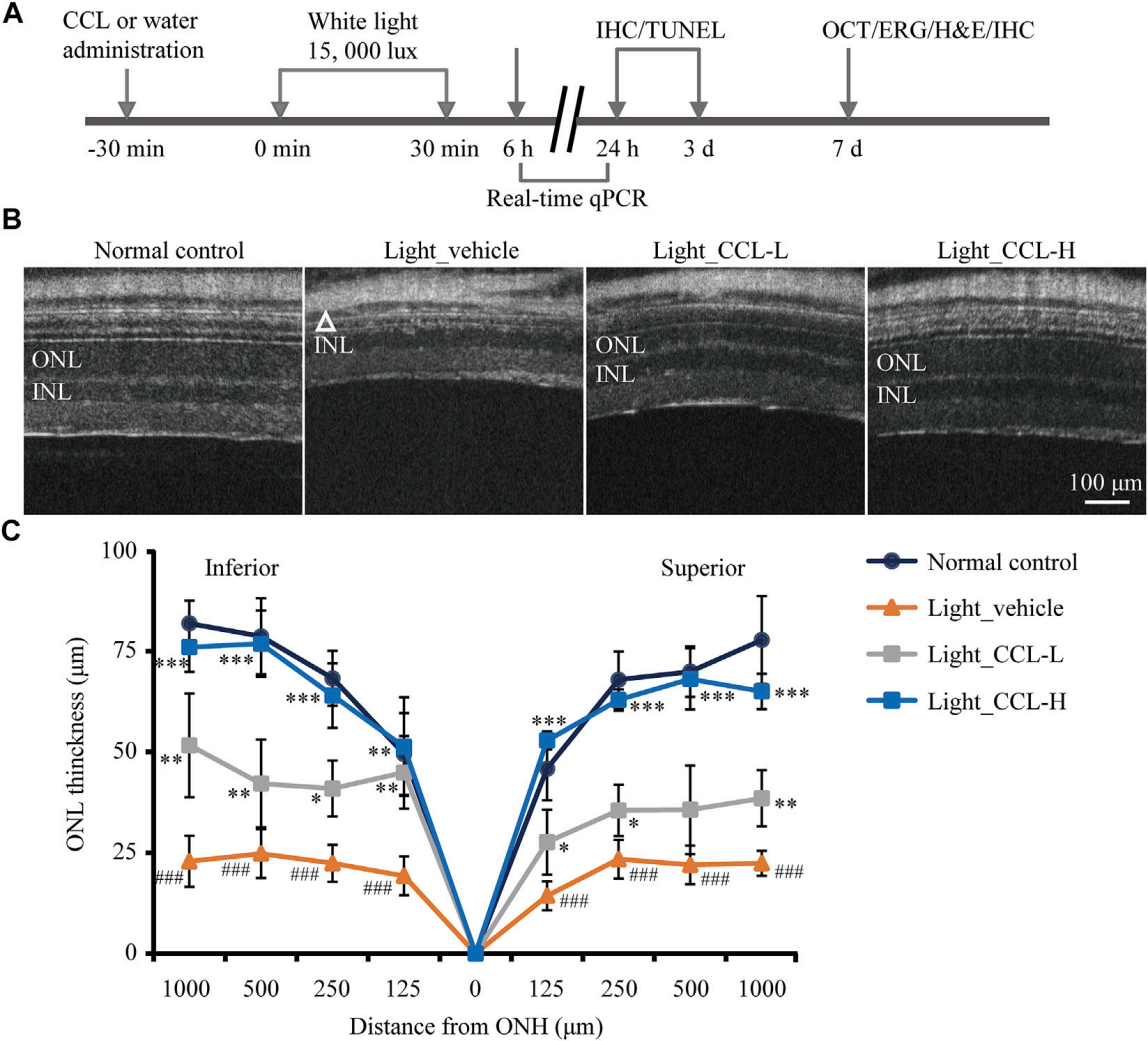
CCL protects against light-induced impairment of the photoreceptor structure. **(A)** The schematic drawing of the experimental design. BALB/c mice were pretreated with CCL or water. Thirty min later, the mice were exposed to white light at 15,000 lux for 30 min, followed by the indicated assessments. **(B)** OCT imaging was carried out 7 days after light exposure to obtain the full-retinal structural images. **(C)** ONL thickness was measured at 125, 250, 500 and 1,000 μm off ONH in the inferior and superior retinas. The white triangle signifies the damaged ONL. INL, inner nuclear layer; ONH, optic nerve head; ONL, outer nuclear layer. Scale bar, 100 μm. Data were expressed as mean ± SD (n = 5 per group). ^###^ Compared to Normal control, *p* < 0.001; * compared to Light_vehicle, *p* < 0.05; ** compared to Light_vehicle, *p* < 0.01; *** compared to Light_vehicle, *p* < 0.001.

Next, the pharmacological potentials of CCL in protecting against photoreceptor degeneration were assessed by multiple experimental approaches in the light-exposed BALB/c mice (Figure 2A). Bright light-induced photoreceptor degeneration is characterized by a gradual loss of photoreceptor structure that physically stabilizes 7 days after illumination (Wenzel et al., 2005). Meanwhile, impairment of the second-order retinal neurons such as bipolar cells and horizontal cells as well as reactive gliosis in müller cells are hallmark pathologies secondary to the degenerative changes in photoreceptors, which persist into the end-stage photoreceptor degeneration and collectively contribute to irreversible deterioration of the retinal structure and function (Chen et al., 2016; Pfeiffer et al., 2020). Thus, the impact of CCL treatment on light-induced structural, functional and morphological impairment of photoreceptors and the pathological alterations in bipolar cells, horizontal cells and müller cells were evaluated by OCT, ERG, H&E staining and IHC assessments 7 days after the light exposure. In addition, our previous studies have demonstrated that microglial activation is most notable prior to the eventual loss of photoreceptor structure (Bian et al., 2016; Wu et al., 2020). IHC examination of microglial activation was thus assessed 24 h and 3 days after light exposure. Furthermore, findings from our previous work indicate that photoreceptor cell death becomes evident 24 h after light exposure and peaks around 3 days after illumination (Bian et al., 2016). Therefore, TUNEL assay was performed 3 days after the light exposure to assess the effect of CCL treatment on photoreceptor cell death. Lastly, it has been shown in our previous studies that the retinal expression of genes involved in inflammatory responses and reactive gliosis was altered at the early stage of photoreceptor degeneration (Bian et al., 2016; Wu et al., 2020), real-time qPCR was thus performed 6 h and/or 24 h after light exposure to evaluate the impact of CCL treatment on the retinal expression of genes implicated in these processes.

To evaluate the photoreceptor protective effects of CCL, BALB/c mice were exposed to experimental white light at the intensity of 15,000 lux for 30 min and treated by vehicle or CCL at 3 g/kg (low-dose CCL) and 12 g/kg bw (high-dose CCL). Non-invasive full-retinal OCT imaging was carried out 7 days after light exposure to obtain an objective and comprehensive view of the retinal structure. As shown in Figure 2B, compared to the vehicle-treated normal controls, the ONL consisting of the cell bodies of rod and cone photoreceptors was evidently diminished in the light-exposed vehicle-treated mice. However, the ONL structure was partial preserved in the light-exposed mice treated with low-dose CCL, whereas nearly complete preservation of the ONL structure was observed in the light-exposed mice treated with high-dose CCL. Measurement of the ONL thickness revealed that the ONL thickness was significantly decreased across the retina in the light-exposed vehicle-treated mice compared to that from the vehicle-treated normal controls (*p* < 0.001). Significantly increased ONL thickness was observed as a result of the low-dose CCL (*p* < 0.05 or 0.01) and high-dose CCL treatment (*p* < 0.01 or 0.001) compared to that from the light-exposed vehicle-treated mice, with greater effect observed in the light-exposed high-dose CCL-treated mice (Figure 2C). The results from OCT imaging indicate that CCL protects photoreceptor against light-induced structural impairment.

### CCL Protects Against Light-Induced Functional Deterioration in the Retina

**FIGURE 3:**
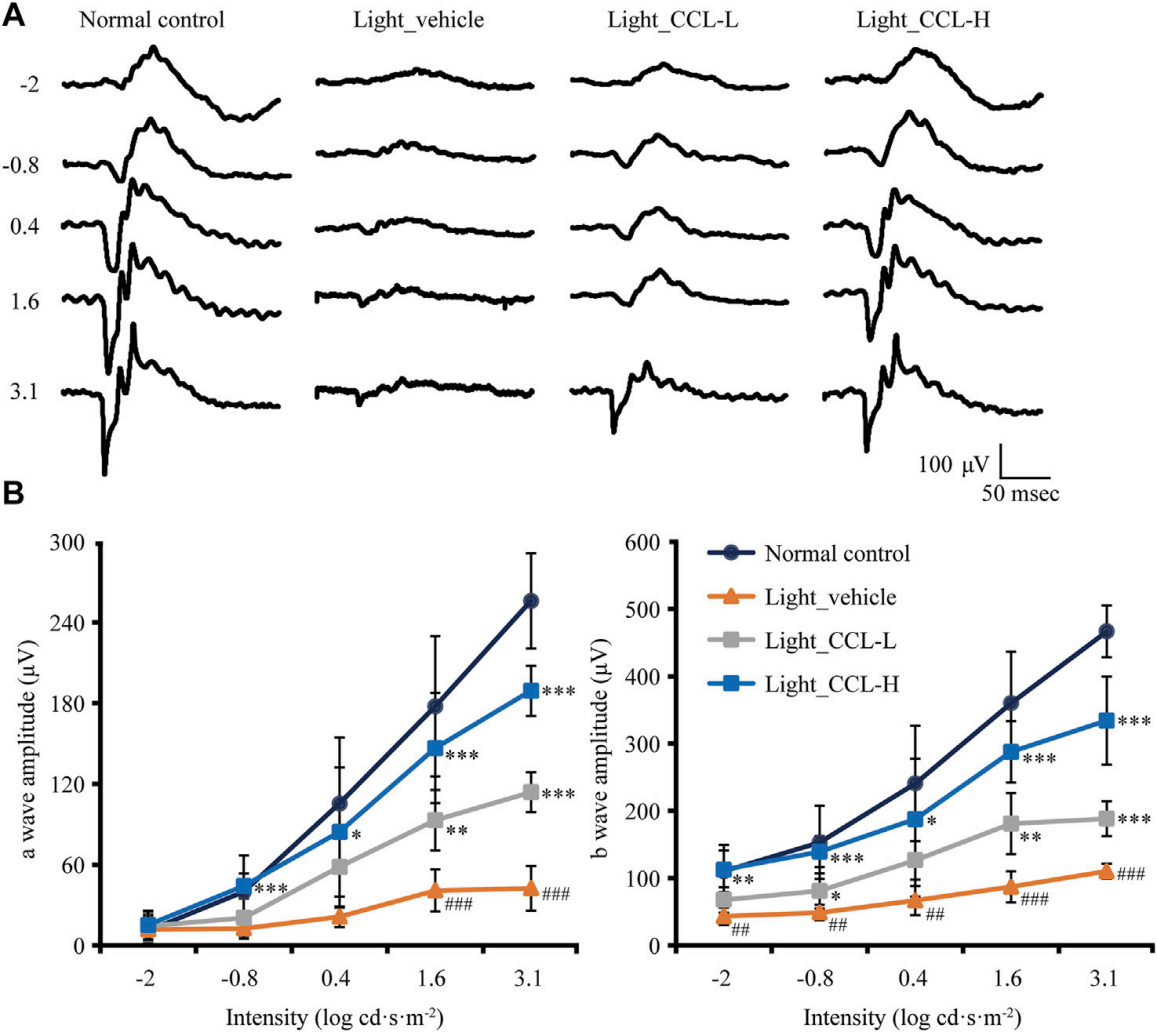
CCL maintains the retinal function in the light-exposed mice. Scotopic ERG was performed 7 days after light exposure to assess the retinal function. **(A)** Representative electroretinograms from the indicated experimental groups. **(B)** Amplitudes of a wave and b wave were plotted. Data were expressed as mean ± SD (n = four to five per group). ^#^ Compared to NC, *p* < 0.05; ^##^ Compared to Normal control, *p* < 0.01; ^###^ compared to NC, *p* < 0.001; * compared to Light_vehicle, *p* < 0.05; ** compared to Light_vehicle, *p* < 0.01; *** compared to Light_vehicle, *p* < 0.001.

Next, full-retinal ERG recording was performed to evaluate the effect of CCL treatment on the scotopic a wave and b wave, which reflect the retinal function conducted by rod photoreceptors and second-order neurons, respectively. As shown in Figure 3A, compared to light-evoked responses in a wave and b wave in the vehicle-treated normal controls, depressed a wave and b wave responses to light stimuli were readily observed in the light-exposed vehicle-treated mice. In contrast, a wave and b wave responses to the light stimuli were improved in the light-exposed mice treated with both low-dose and high-dose CCL. Quantification of a wave and b wave amplitudes revealed significantly decreased a wave and b wave amplitudes in the light-exposed vehicle-treated retinas compared to the vehicle-treated normal controls (a wave: *p* < 0.001; b wave: *p* < 0.01 or 0.001). Both low-dose and high-dose CCL treatment resulted in increased a wave and b wave amplitudes compared to that from the light-exposed vehicle-treated mice (a wave: *p* < 0.05, 0.01 or 0.001; b wave: *p* < 0.05, 0.01 or 0.001), with better effects observed in the light-exposed high-dose CCL-treated mice (Figure 3B). These results indicate that CCL treatment protects against light-induced impairment of the retinal function.

### CCL Confers Protection to Rod and Cone Photoreceptors in the Light-Exposed Retinas

**FIGURE 4:**
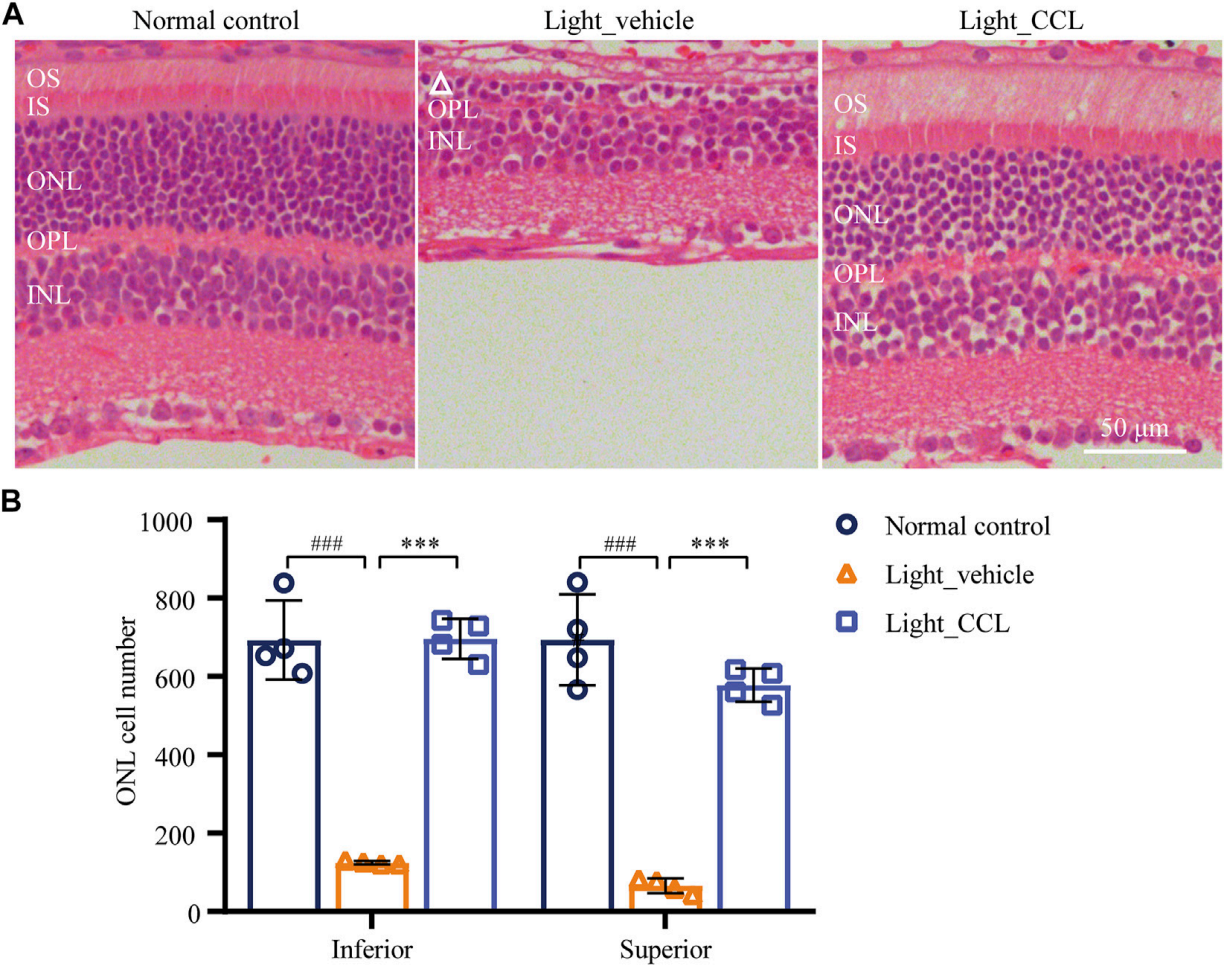
CCL protects against light-induced morphological damage to the photoreceptors. Histological examination was performed by H&E staining. **(A)** Representative micrographs from the indicated experimental groups. **(B)** The number of photoreceptors was counted from ONH to 400 μm off ONH in the inferior and superior retinas. White triangle signifies the damage ONL. INL, inner nuclear layer; IS, inner segment; ONH, optic nerve head; ONL, outer nuclear layer; OPL, outer plexiform layer; OS, outer segment. Scale bar, 50 μm. The data were expressed as mean ± SD (n = 4 per group). ^###^ Compared to Normal control, *p* < 0.001; *** compared to Light_vehicle, *p* < 0.001.

**FIGURE 5:**
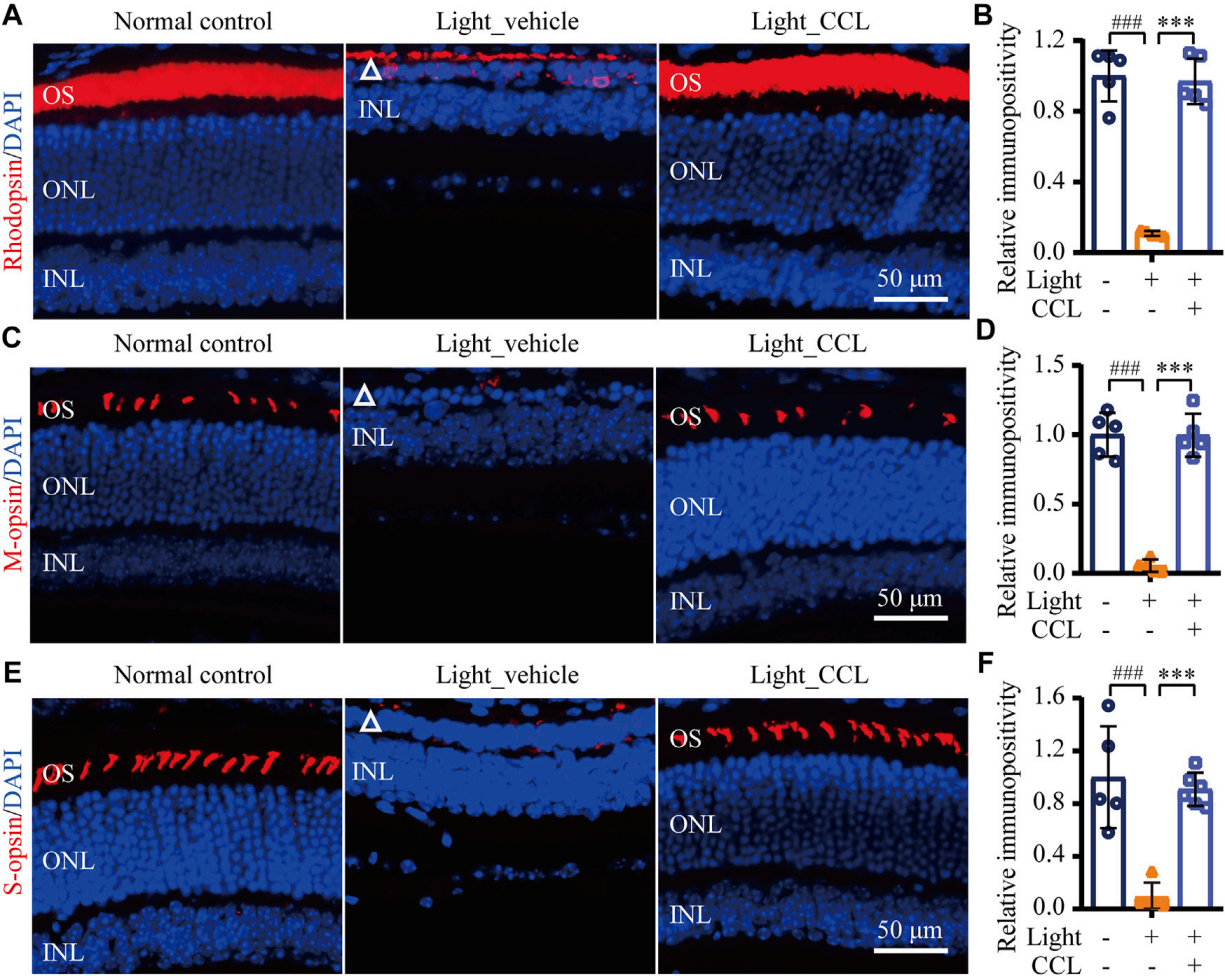
CCL protects against light-induced ablation of rod and cone photoreceptors. IHC was performed to examine the expression of rhodopsin, M-opsin and S-opsin in the retina (in red). DAPI counterstaining (in blue) was performed to visualize the nuclei. The immunopositivity of rhodopsin, S-opsin and M-opsin in photoreceptor OS was quantified using ImageJ. **(A)** Representative micrographs showing the expression of rhodopsin in the retina. **(B)** The relative fold change in rhodopsin immunopositivity was plotted against that from Normal control. **(C)** Representative micrographs showing the expression of S-opsin in the retina. **(D)** The relative fold change in the immunopositivity of S-opsin was plotted against that from Normal control. **(E)** Representative micrographs showing the expression of M-opsin in the retina. **(F)** The relative fold change in the immunopositivity of M-opsin was plotted against that from Normal control. White triangle points to damaged ONL. INL, inner nuclear layer; ONL, outer nuclear layer; OS, outer segment. Scale bar, 50 μm. The data were expressed as mean ± SD (n = 5 per group). ^###^ Compared to Normal control, *p* < 0.001; *** compared to Light_vehicle, *p* < 0.001.

As demonstrated above, high-dose CCL treatment resulted in nearly complete protection of the photoreceptor structure and retinal function in the light-exposed mice. Therefore, detailed histological and immunohistochemical examinations were carried out to further characterize the protective effective of high-dose CCL (marked as CCL in the following experiments) on the light-exposed retinas. First, H&E staining was performed to gain a better visualization of the gross retinal morphology. As shown in Figure 4A, compared to the vehicle-treated normal controls, photoreceptor outer segment (OS) and inner segment (IS) were nearly diminished and the ONL was severely damaged in the vehicle-treated light-exposed mice. However, the morphology of OS, IS and ONL was preserved in the light-exposed mice treated with CCL. Measurement of the number of nuclei in ONL demonstrated a significant reduction in the number of photoreceptor cell bodies in both inferior and superior retinas from the light-exposed vehicle-treated mice compared to the vehicle-treated normal controls (*p* < 0.001 in both the inferior and superior retinas), whereas the number of nuclei in ONL in the inferior and superior retinas was increased in the light-exposed CCL-treated mice compared to that from the light-exposed vehicle-treated mice (*p* < 0.001 in both the inferior and superior retinas) (Figure 4B). Next, to better visualize the protection of rod and cone photoreceptors conferred by CCL treatment, IHC was performed to specifically label rods, short wavelength-sensitive cones and mid wavelength-sensitive cones in the retina. As shown in Figures 5A,B, compared to the abundantly expressed rod-specific rhodopsin in the retinas from the vehicle-treated normal controls, only residual expression of rhodopsin was detected in the retinas from the light-exposed vehicle-treated mice (*p* < 0.001). In contrast, the expression of rhodopsin was well preserved in the light-exposed CCL-treated retinas compared to that from the light-exposed vehicle-treated mice (*p* < 0.001). Similarly, the retinal expression of short wavelength-sensitive cone-specific S-opsin and mid wavelength-sensitive cone-specific M-opsin was found to be nearly diminished in the light-exposed vehicle-treated retinas (*p* < 0.001), whereas the expression of both S-opsin (Figures 5C,D) and M-opsin (Figures 5E,F) was preserved in the light-exposed CCL-treated retinas (*p* < 0.001). These results collectively demonstrate that CCL treatment protects rod and cone photoreceptors against light-induced morphological damage.

### CCL Maintains the Integrity of the Second-Order Neurons in the Light-Exposed Retinas

**FIGURE 6:**
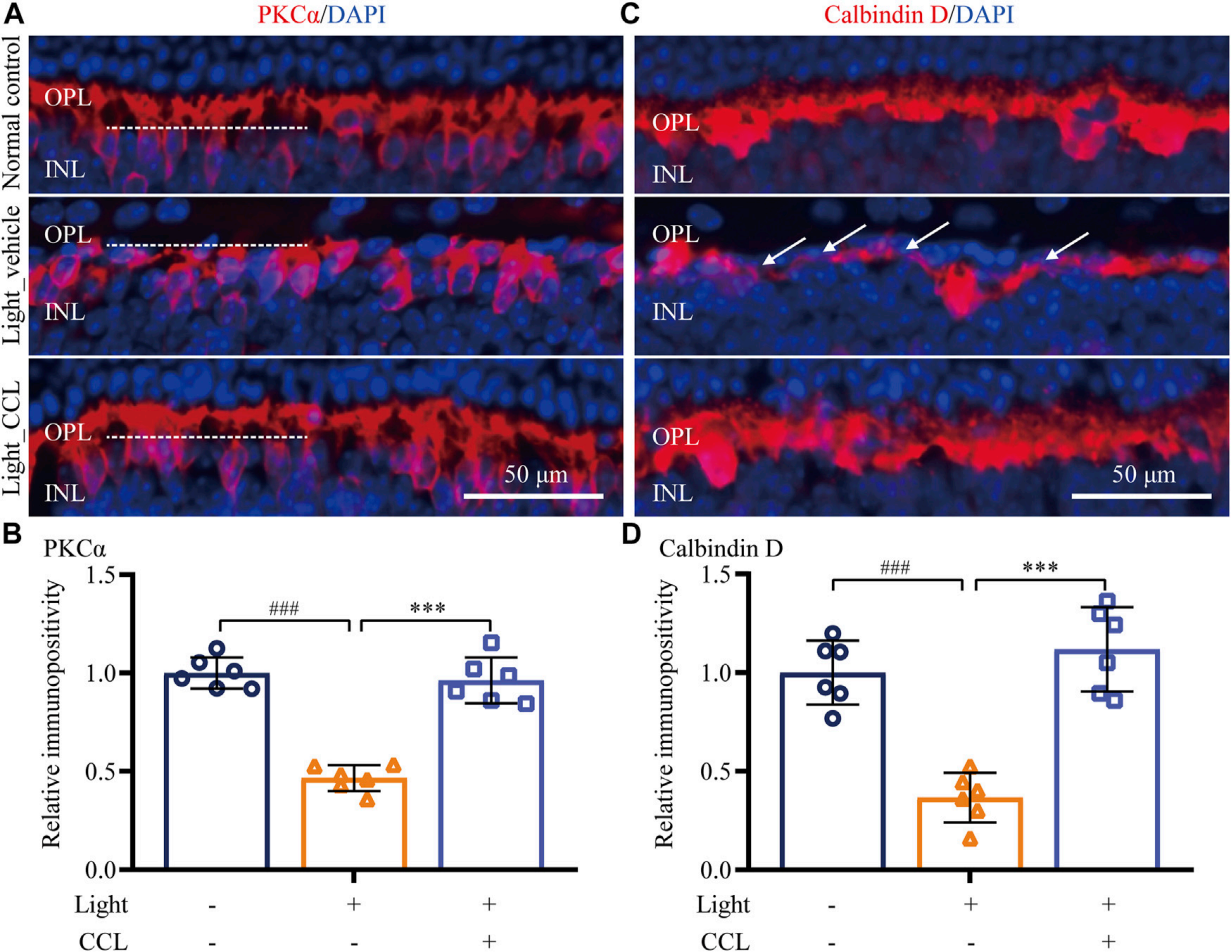
CCL attenuates the morphological impairment of bipolar and horizontal cells in the light-exposed retinas. IHC was performed to examine the expression of PKCα (in red) and calbindin D (in red) in the retina. Counterstaining of DAPI (in blue) was performed. The immunopositivity of PKCα and calbindin D in OPL was quantified by ImageJ. **(A)** Representative micrographs showing the expression of PKCα in the retina. **(B)** The relative fold change in the immunopositivity of PKCα was plotted against that from Normal control. **(C)** Representative micrographs showing the expression of calbindin D in the retina. **(D)** The relative fold change in the immunopositivity of calbindin D was plotted against that from Normal control. INL, inner nuclear layer; OPL, outer plexiform layer. Scale bar, 50 μm. Data were expressed as mean ± SD (n = 6 per group). ^###^ Compared to Normal control, *p* < 0.001; *** compared to Light_vehicle, *p* < 0.001.

Photoreceptor degeneration often leads to impairment of second-order retinal neurons, which contributes to the functional deterioration of the visual processing pathway. Thus, the effect of CCL treatment on the integrity of the second-order retinal neurons, bipolar cells and horizontal cells, were further examined. As shown in Figures 6A,B, PKCα labeling revealed that the dendrites of rod bipolar cells were impaired in the retinas from the light-exposed vehicle-treated mice (*p* < 0.001). However, the dendritic morphology of the bipolar cells was preserved in the retinas from the light-exposed CCL-treated mice (*p* < 0.001). Moreover, calbindin D labeling showed that compared to the vehicle-treated normal controls, the dendrites of horizontal cells were evidently damaged in the light-exposed vehicle-treated mice (*p* < 0.001). In sharp contrast, the morphological integrity of horizontal cell dendrites was preserved in the light-exposed CCL-treated mice (*p* < 0.001) (Figures 6C,D). These results demonstrate that CCL confers remarkable protection to the second-order neurons in the light-exposed retinas.

### CCL Attenuates Photoreceptor Cell Death in the Light-Exposed Retinas

**FIGURE 7:**
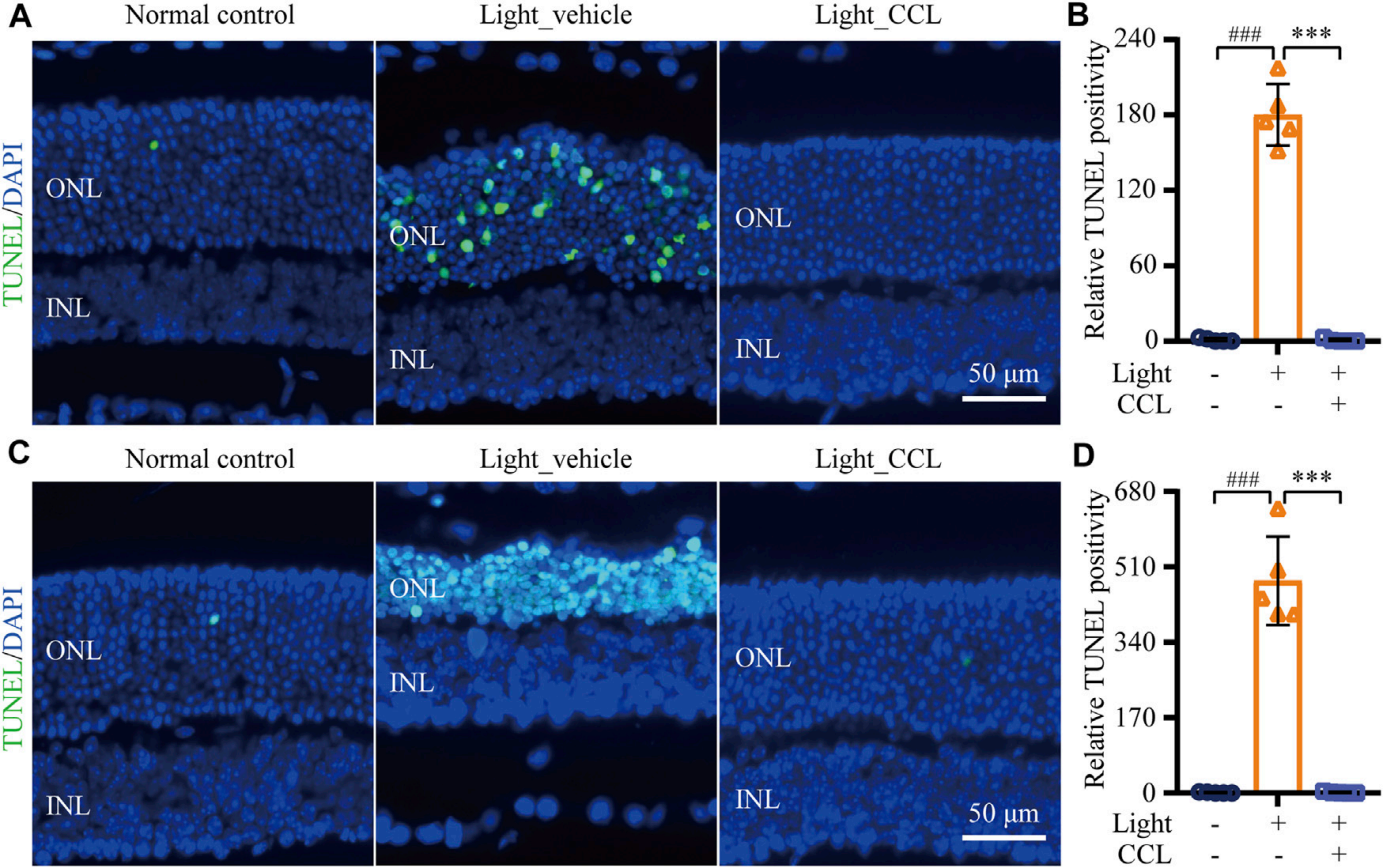
CCL suppresses photoreceptor cell death in the light-exposed retinas. TUNEL assay was performed to assess photoreceptor cell death. Nuclei were stained by DAPI (in blue). TUNEL positivity (in green) in ONL was quantified by ImageJ. **(A)** Representative micrographs demonstrating TUNEL positivity from the eyes enucleated 24 h after light exposure. **(B)** The relative fold change in the TUNEL positivity from **(A)** was plotted against that from Normal control. **(C)** Representative micrographs demonstrating TUNEL positivity from the eyes enucleated 3 days after light exposure. **(D)** The relative fold change in the TUNEL positivity from **(C)** was plotted against that from Normal control. INL, inner nuclear layer, ONL, outer nuclear layer. Scale bar, 50 μm. Data were expressed as mean ± SD (n = 5 per group). ^###^ Compared to Normal control, *p* < 0.001; *** compared to Light_vehicle, *p* < 0.001.

Cell death is the central cellular mechanism that directly leads to loss of the photoreceptor structure and function. Our previous study has demonstrated a progressive increase in photoreceptor cell death prior to the eventual loss of photoreceptor structure (Bian et al., 2016). To further understand the impact of CCL treatment on photoreceptor cell death in the light-exposed retinas, TUNEL assay was performed. As shown in Figure 7, TUNEL positive cells were rare in the retinas from the vehicle-treated normal controls. However, a visible increase in TUNEL positivity was readily observed in the ONL from the light-exposed vehicle-treated mice 1 day (Figures 7A,B) and 3 days (Figures 7C,D) after the light exposure (*p* < 0.001). In distinct contrast, TUNEL positivity was barely observed in the retinas from the light-exposed CCL-treated mice at both time points (*p* < 0.001). These results indicate that CCL treatment suppresses photoreceptor cell death in the light-exposed retinas.

### CCL Alleviates Müller Cell Gliosis in the Light-Exposed Retinas

**FIGURE 8:**
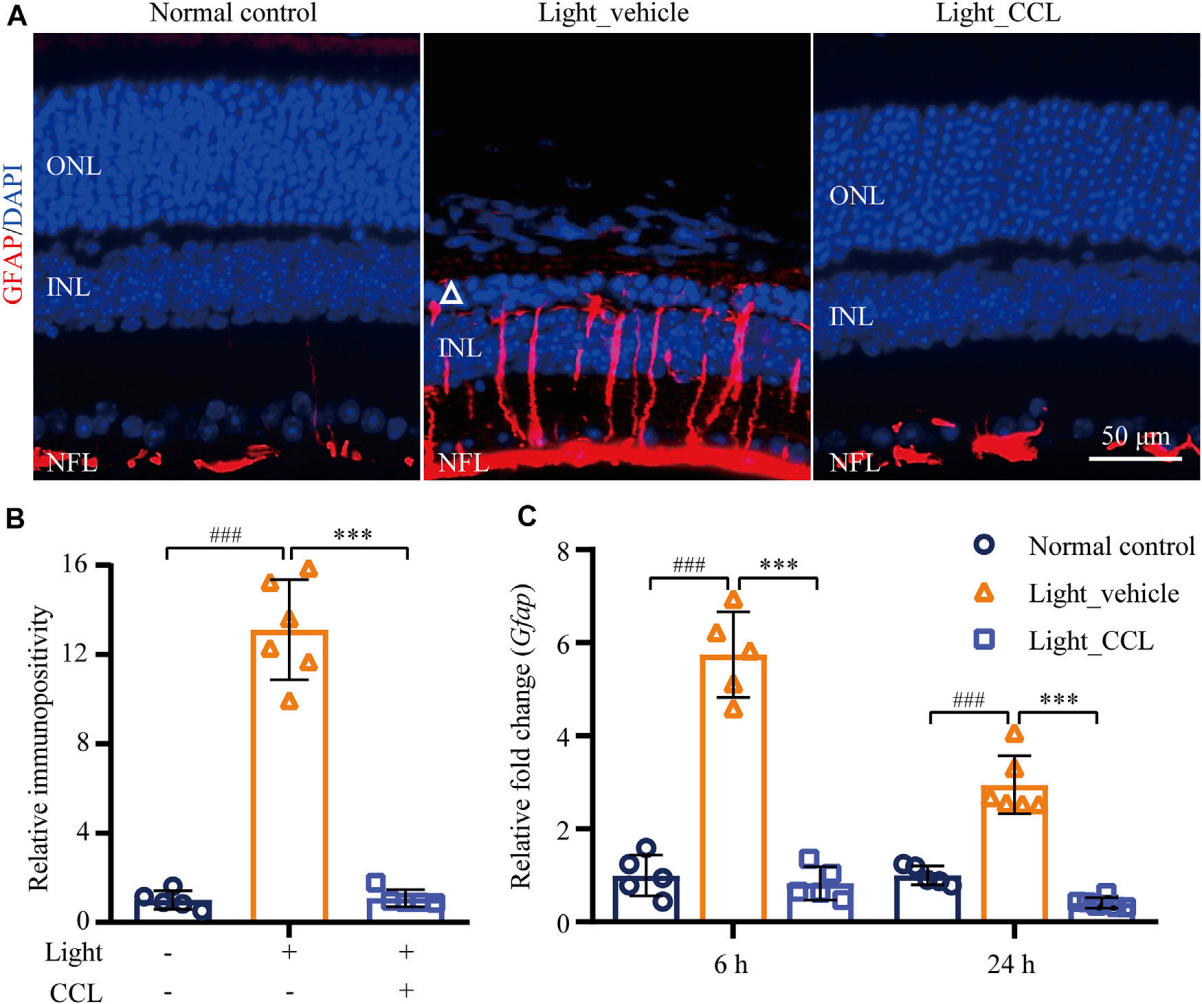
CCL attenuates reactive müller gliosis in the light-exposed retinas. IHC was performed to examine the expression of GFAP in the retina (in red). Counterstaining of DAPI (in blue) was performed. The immunopositivity of GFAP in the retina was quantified by ImageJ. **(A)** Representative micrographs showing the expression of GFAP in the retina. **(B)** The relative immunopositivity of GFAP in the indicated experimental groups was plotted against that from Normal control. **(C)** Total RNA was isolated from the retinas collected from the indicated experimental groups 6 and 24 h after light exposure. Real-time qPCR was then performed to analyze the retinal expression of GFAP. 18S rRNA was amplified as an internal control. The relative fold change in the expression of GFAP was plotted against that from Normal control. INL, inner nuclear layer; NFL, nerve fiber layer; ONL, outer nuclear layer. Scale bar, 50 μm. Data were expressed as mean ± SD (n = 5-6 per group). ^###^ Compared to Normal control, *p* < 0.001; *** compared to Light_vehicle, *p* < 0.001.

Photoreceptor degeneration is often accompanied by excessive müller cell gliosis. The müller cell gliosis is not only a sensitive pathological alteration signifying even subtle disturbance of the retinal homeostasis, it may further aggravates retinal degeneration (Dyer and Cepko, 2000). The retinal expression of GFAP was thus evaluated to assess the effect of CCL on the reactive changes in the müller cells in the light-exposed retinas. As shown in Figure 8A, GFAP positivity was primarily restricted to the nerve fiber layer (NFL) in the retinas from the vehicle-treated normal controls. In distinct contrast, the GFAP positivity was readily detected not only in the NFL, but also in the inner plexiform layer (IPL), inner nuclear layer (INL) and ONL in the light-exposed vehicle-treated retinas 7 days after the experimental light exposure, indicative of müller gliosis accompanying photoreceptor degeneration. However, this aberrancy in the GFAP expression pattern was attenuated in the light-exposed CCL-treated retinas. Quantification of the GFAP positivity consistently revealed significantly increased GFAP immunopositivity in the light-exposed vehicle-treated retinas compared to that from the vehicle-treated normal controls (*p* < 0.001). Compared to that from the light-exposed vehicle-treated retinas, GFAP immunopositivity was markedly reduced as a result of the CCL treatment (Figure 8B) (*p* < 0.001). In addition, real-time qPCR was performed to analyze the mRNA level of GFAP in the retina. As shown in Figure 8C, significantly increased mRNA expression of GFAP was detected in the light-exposed vehicle-treated retinas as early as 6 h (*p* < 0.001) and 24 h (*p* < 0.001) after the light exposure. In sharp contrast, the mRNA level of GFAP was significantly decreased in the light-exposed CCL-treated retinas compared to that from the light-exposed vehicle-treated retinas (*p* < 0.001 at both 6 and 24 h). These results demonstrate that CCL treatment suppresses light-induced reactive müller gliosis in the retinas.

### CCL Suppresses Microglial Activation and Inflammatory Responses in the Light-Exposed Retinas

**FIGURE 9:**
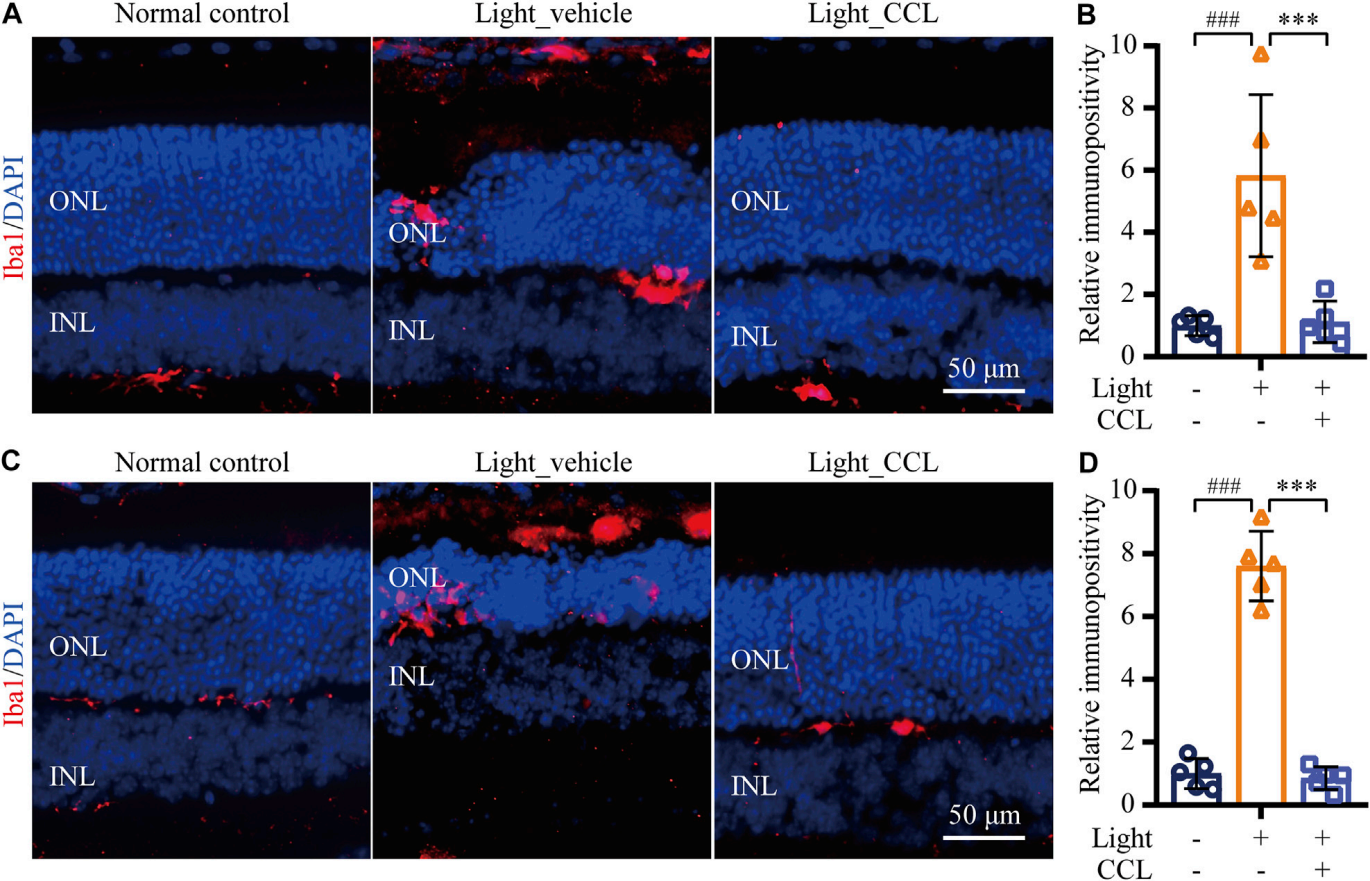
CCL mitigates microglial activation in the light-exposed retinas. IHC was performed to examine the expression of Iba-1 in the retina (in red). Counterstaining of DAPI (in blue) was performed. The immunopositivity of Iba-1 in the outer retina including ONL and the subretinal space was quantified by ImageJ. **(A)** Representative micrographs demonstrating Iba-1 positivity from the eyes enucleated 24 h after light exposure. **(B)** The relative fold change in the immunopositivity of Iba-1 from **(A)** was plotted against that from Normal control. **(C)** Representative micrographs demonstrating Iba-1 positivity from the eyes enucleated 3 days after light exposure. **(D)** The relative fold change in the immunopositivity of Iba-1 from **(C)** was plotted against that from Normal control. INL, inner nuclear layer; ONL, outer nuclear layer. Scale bar, 50 μm. Data were expressed as mean ± SD (n = 5 per group). ^###^ Compared to Normal control, *p* < 0.001; *** compared to Light_vehicle, *p* < 0.001.

**FIGURE 10:**
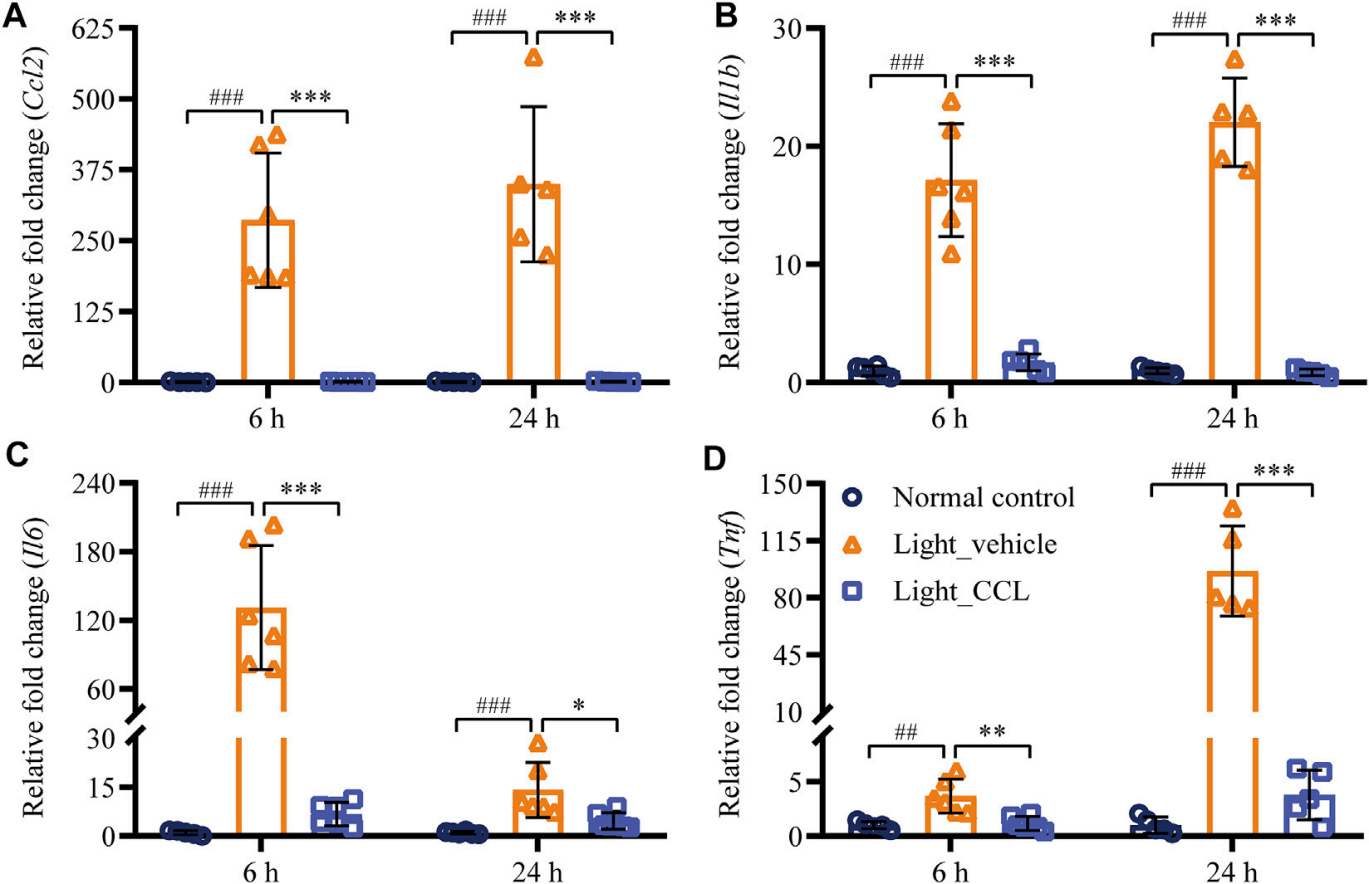
CCL suppresses the expression of proinflammatory genes in the light-exposed retinas. Total RNA was isolated from the retinas collected from the indicated experimental groups 6 and 24 h after light exposure. Real-time qPCR analyses were performed to examine the expression of *Ccl2* **(A)**, *Il1b* **(B)**, *Il6* **(C)**, and *Tnf* **(D)** in the retina. Relative fold change was normalized against that from Normal control. The data were expressed as the mean ± SD (n = 5-6 per group). ^##^ Compared to Normal control, *p* < 0.01; ^###^ Compared to Normal control, *p* < 0.001; * compared to Light_vehicle, *p* < 0.05; ** compared to Light_vehicle, *p* < 0.01; *** compared to Light_vehicle, *p* < 0.001.

Following photoreceptor degeneration, resident microglial cells are activated to clear the damaged retinal neurons. However, uncontrolled microglial activation exacerbates neuronal cell death in the damaged retina through excessive inflammatory responses (Xu et al., 2009). To evaluate the effect of CCL treatment on photoreceptor degeneration-associated microglial activation in the retina, the retinal expression of the microglial marker Iba-1 was examined. The results showed that Iba-1 positivity was increased in the light-exposed vehicle-treated retinas 1 day (*p* < 0.001) (Figures 9A,B) and 3 days (*p* < 0.001) (Figures 9C,D) after the light exposure, whereas much less Iba-1 positivity was observed in the light-exposed CCL-treated retinas at both time points (*p* < 0.001). Furthermore, the retinal expression of genes encoding inflammation mediators including Ccl2, Il1b, Il6, and Tnf was further analyzed. As shown in Figure 10, compared to the vehicle-treated normal controls, significantly increased retinal expression of *Ccl2* (*p* < 0.001), *Il1b* (*p* < 0.001), *Il6* (*p* < 0.001) and *Tnf* (*p* < 0.01) was observed in the light-exposed vehicle-treated mice 6 h after the light exposure. In distinct contrast, significantly decreased expression of *Ccl2* (*p* < 0.001), *Il1b* (*p* < 0.001), *Il6* (*p* < 0.001) and *Tnf* (*p* < 0.01) was noted in the light-exposed CCL-treated retinas 6 h after the light exposure. Similar results were observed 24 h after light exposure. Compared to the vehicle-treated normal controls, increased retinal expression of *Ccl2* (*p* < 0.001), *Il1b* (*p* < 0.001), *Il6* (*p* < 0.001), and *Tnf* (*p* < 0.001) was observed in the light-exposed vehicle-treated mice 24 h after the light exposure. However, significantly decreased expression of *Ccl2* (*p* < 0.001), *Il1b* (*p* < 0.001), *Il6* (*p* < 0.05), and *Tnf* (*p* < 0.001) was noted in the light-exposed CCL-treated retinas. These results collectively indicate that CCL treatment alleviates photoreceptor degeneration-associated microglial activation and inflammatory responses in the retina.

## Discussion

The current study demonstrates that CCL provides structural, morphological and functional protection against light-induced photooxidative stress-mediated photoreceptor degeneration. These photoreceptor protective effects of CCL are in part attributable to inhibition of photoreceptor cell death, preservation of the morphological integrity of the second-order retinal neurons as well as mitigation of müller gliosis, microglial activation and retinal inflammation. Thus, the findings here provide direct evidence supporting the protective effects of CCL on photoreceptors and the retinal microenvironment under photooxidative stress conditions.

The major findings from the current work relate to photoreceptor protection conferred by CCL. CCL has been used for the treatment of a broad range of disorders including reproductive disorders, hepatic diseases, renal diseases, aging and ophthalmic diseases affecting vision (Zheng HZD and She, 1998; Donnapee et al., 2014; Zhu et al., 2020). The traditional views on the vision-enhancing effects of CCL can be found in the earliest Chinese pharmacopoeia, *Shennong’s Herbal* as well as the classic work of Chinese materia medica, *Compendium of Materia Medica*. In terms of the ophthalmic diseases, CCL has been included as one of the major components in various TCM formulas to treat not only degenerative retinal diseases such as dry AMD, RP, glaucoma and ischemic optic neuropathy, but also neovascular retinal disorders, for instance, wet AMD and diabetic retinopathy. In addition, CCL-containing TCM formulas have also been widely used to treat cataract and dry eye syndrome, disease entities that affect the conjunctiva and lens (Zhu et al., 2020). The reproductive effects, hepatoprotective, renoprotective, cardioprotective, anti-aging, neuroprotective and anti-diabetic activities of CCL have been supported by multiple lines of pharmacological studies (Donnapee et al., 2014). Our previous study has also demonstrated that a CCL-containing TCM formula, Shihu Yeguang Pill, protects against light-induced photoreceptor degeneration (Wu et al., 2020). However, to our best knowledge, the pharmacological implications of CCL *per se* in photoreceptor degenerative diseases have not been addressed. Here, by demonstrating that CCL counteracts light-induced impairment of the photoreceptor structure and associated retinal function, our study provides direct *in vivo* experimental evidence that supports the photoreceptor protective properties of CCL, thereby justifying the clinical application of CCL in the prevention and treatment of photoreceptor degenerative diseases such as dry AMD and RP.

As an integral part of the central nervous system, the light-sensing retina conducts the essential function of vision formation (Demb and Singer, 2015). It contains millions of light-sensitive first-order neurons, namely, photoreceptors as well as the second-order and the third-order neurons. Photoreceptors, the first-order retinal neurons, are the primary sensory neurons in the retina. They capture and convert the incoming light into an electrical signal that is further processed through the second-order and the third-order retinal neurons. The electrical visual signal initially generated in the photoreceptors is eventually carried to the brain to create conscious vision. There are two types of photoreceptors, namely rods and cones. Rods are extremely sensitive and carry out specialized function in mediating low-light vision. Cones are responsible for the generation of daylight and color vision. Like other neurons in the central nervous system, photoreceptors are postmitotic and the loss of photoreceptors is irreversible, which directly leads to vision impairment in the patients with related retinal degenerative conditions such as AMD and RP (Hoon et al., 2014). The photoreceptors are exposed to high concentration of oxygen from choriocapillaries (Wangsa-Wirawan and Linsenmeier, 2003). Photoreceptor OS is rich in lipids and is vulnerable to oxidation. Light exposure, oxidization of polyunsaturated fatty acids, and phagocytosis of photoreceptor OS routinely produce high levels of free radicals (Fliesler and Bretillon, 2010). Therefore, even under physiological conditions, photoreceptors are under constant oxidative stress. Under pathological conditions, whether caused by genetic mutation/deficiency or environmental insults, oxidative stress is regarded as the major mechanism mediating multiple biochemical and molecular changes that eventually lead to the death and degeneration of the stressed photoreceptors (Domènech and Marfany, 2020). Bright light exposure creates excessive photo-oxidative stress to the retina and thus has been extensively used to recapitulate oxidative stress-induced photoreceptor degeneration (Organisciak et al., 1998; Wenzel et al., 2005). Our work here demonstrates that CCL attenuates bright light-induced photoreceptor cell death and preserves the structure and function integrity of the retina in the bright light-exposed mice (Figures 2–7), supporting the notion that CCL attenuates oxidative stress-mediated photoreceptor degeneration. Future studies are worth pursuing to further elucidate the molecular mechanisms underlying the anti-oxidant effects of CCL in the pathological context of photoreceptor degeneration.

Secondly, our work here demonstrates that other than significant protection over photoreceptors, CCL treatment maintains the morphological integrity of the second-order bipolar and horizontal cells under photooxidative stress conditions (Figure 6). Bipolar cells and horizontal cells are the essential components of the retinal neuronal network (Euler et al., 2014). Operating in the similar fashion as that of the brain, the function of the neuronal network of the retina is also dependent on the spatially ordered organization of projection neurons and interneurons as well as their synaptic connections. Photoreceptors form synaptic connections with bipolar cells and horizontal cells, constituting the first information relay point in the visual pathway (Burger et al., 2021). Bipolar cells are the projection neurons in the retina, relaying the information generated in photoreceptors to the third-order retinal ganglion cells, the specialized retinal neurons that send out the vision information to the brain. Given that bipolar cells are in charge of relaying the information for vision formation, they constitute the elementary building blocks of vision. Horizontal cells are the interneurons in the retina, forming triad synapses with photoreceptors and bipolar cells. Horizontal cells function to modulate signal transmission from photoreceptors to bipolar cells (Thoreson and Mangel, 2012). Ablation of horizontal cells leads to morphological degeneration of rod photoreceptors and impaired retinal function (Sonntag et al., 2012). Therefore, maintaining the morphological integrity of bipolar cells and horizontal cells contribute to the protective effects on the retinal structure and function as a result of CCL treatment. However, whether CCL provides direct protection to bipolar and/or horizontal cells requires further investigation in the future studies.

In addition, CCL treatment results in a remarkable attenuation of the gliotic changes in the müller cells in the light-exposed retinas (Figure 8). Müller cells are macroglial cells in the retina that structurally support the retinal neurons. Meanwhile, they are held responsible for maintaining the homeostasis of the neuronal microenvironment in the retina (Bringmann et al., 2006). However, under pathological conditions, instead of being neuronal supportive, müller cells experience reactive gliotic changes and develop gliotic scars that may detrimentally disturb the retinal homeostasis and further exacerbate neuronal cell death. Thus, reactive müller gliosis is not only one of the most sensitive hallmark pathologies associated with nearly all known retinal disorders, it also promotes the progression of neuronal degeneration by enhancing the susceptibility of retinal neurons to stress stimuli (Bringmann et al., 2006). Considering the important implications of müller cells in the retinal homeostasis, suppressed müller gliosis may in part contribute to the photoreceptor protection observed in the light-exposed CCL-treated mice. The direct impact of CCL treatment on reactive müller gliosis, however, requires further delineation.

Lastly, our work here demonstrates that CCL treatment attenuates microglial activation and inflammatory gene expression in the light-exposed retinas. It is a well-established notion that enhanced inflammatory response exacerbates retinal cell death (Xu et al., 2009; Anderson et al., 2002; Hollborn et al., 2008). The activated microglial cells promote inflammatory responses and aggravate cell death in the neuronal tissues. Accumulated evidence has revealed that microglial activation is not merely a common pathology associated with the degenerated retinas, it is also neurotoxic and aggravates photoreceptor loss. Suppressing microglial activation has been demonstrated to be neuroprotective in light-challenged retinas (Kohno et al., 2013; Scholz et al., 2015). Therefore, microglial activation has emerged as a new target for photoreceptor protective treatment. Our work here demonstrates that CCL treatment suppresses microglial activation as well as the expression of proinflammatory genes in the light-exposed retinas (Figures 9, 10), which may in part contribute to its protective effect against light-induced photoreceptor degeneration. Meanwhile, oxidative stress not only directly leads to cell death, but also triggers inflammatory responses (Ozawa, 2020). Therefore, it is also possible that attenuated microglial activation occurs as a result of CCL-conferred suppression of the photooxidative stress in the retina. Further studies are thus necessary to clarify whether CCL plays any direct roles in suppressing microglial inflammatory activation.

It is also worth noting that quercetin, a type of flavonoids found in CCL, has been shown to mitigate light-induced retinal degeneration in rats (Koyama et al., 2019). The pharmacological potential of quercetin in attenuating neuroinflammation accompanying photoreceptor degeneration has also been demonstrated in a mouse model of RP (Ortega and Jastrzebska, 2021). In addition, it has been reported that kaempferol, another flavonoid component of CCL, protects against oxidizing agent sodium iodate-induced photoreceptor degeneration (Du et al., 2018). These findings provide pharmacological hypotheses in understanding the chemical basis of the retinal protective effects of CCL as revealed by the current work. However, future studies are required to elucidate to what extent quercetin and kaempferol contribute to the effects of CCL in protecting the retina from developing photooxidative stress-mediated photoreceptor degeneration and associated retinal inflammation. Additionally, given that CCL decoction instead of the individual bioactive compound in CCL was analyzed in the current study, our findings here only relate to the photoreceptor protective effects of the total composition of CCL with indicated chemical profiles shown in Figure 1. A synergistic effect of different bioactive compounds in CCL decoction is likely at play in protecting the retina from developing bright light-induced degenerative pathologies. This notion also stresses the standardization of CCL preparation in analyzing its photoreceptor protective activities.

In conclusion, our current work presents novel experimental evidence demonstrating that CCL protects against light-induced oxidative stress-mediated photoreceptor cell death, second-order retinal neuron impairment, reactive müller gliosis, microglial activation and retinal inflammation, which may collectively contribute to CCL-conferred preservation of the structural and functional integrity of the retina. The findings here thus provide direct pharmacological evidence supporting TCM-guided clinical application of CCL in the prevention and treatment of vision-threatening photoreceptor degenerative disorders.

## Data Availability

The original contributions presented in the study are included in the article/Supplementary Material, further inquiries can be directed to the corresponding author.
